# Point of View Telemedicine at Point of Care

**DOI:** 10.7759/cureus.3662

**Published:** 2018-11-30

**Authors:** Wells Weymouth, Lane Thaut, Nathan Olson

**Affiliations:** 1 Emergency Medicine, San Antonio Military Medical Center, San Antonio, USA

**Keywords:** telemedicine, emergency, austere, deployed

## Abstract

Introduction

The use of telemedicine by deployed healthcare providers to improve patient care has been increasing in recent foreign conflicts and humanitarian missions. These efforts have mostly been limited to email consultation with long response lag times. The United States Military has developed several modalities of telemedicine for use in austere environments, ranging from video conferencing, email, and store-and-forward technology. As of now, these efforts have required large pieces of equipment and many technical support personnel and have a delayed response time. Our study aimed to test the overall feasibility of use, the effects on time to intervention, and user confidence in a highly portable, real-time video set-up to aid in teleconsultations at the early stages of care for a simulated traumatic injury.

Materials and methods

Subjects or operators taking direct care of the simulated patient were junior emergency medicine (EM) residents or military trained medics. Video teleconsultation was completed by either senior EM residents in their final year of training or board-certified EM physicians. The subjects taking direct care of the simulated patient were blinded to whether their video device was actively sending images or not. All participants communicated verbally using hand-held radios. The total number of interventions and time to event analysis was completed and survey data were collected, assessing confidence levels on procedures performed and patient care.

Results

We demonstrated the accessibility, ease of use, and overall practicality of this telemedicine platform. A trend was found towards decreased time to evacuation for patients with a live video feed. Alternatively, the data showed no significant difference in the addition of video as opposed to solely radio in terms of the number of interventions, time to interventions, or operator or teleconsultant confidence in the care delivered or procedures performed.

Conclusions

This study demonstrated the overall feasibility and ease of use of a highly portable telemedicine platform with live video capabilities. A trend was found toward earlier evacuation decisions when using the live video. Follow-up studies may consider examining more challenging simulations or prolonged field care utilization of this technology.

## Introduction

The recent US military's worldwide mission has increased to include more remote and austere regions, requiring complex and commonly prolonged medical care provided by medics [1-2]. Increasingly, forward-deployed military healthcare providers have desired to communicate with their extra-theater physician and medical specialists, with the aim of providing improved patient care and outcomes [3]. Generally, these efforts have been limited to email consultations and online X-ray imaging with the exception of a specialized US Army team with the video-conferencing capability [3-4].

Telemedicine is not a new technology; rather, it was largely developed by the National Aeronautics and Space Administration, Lockheed Martin, and the Indian Health Services in the 1970s. These organizations teamed up to provide real-time video, data, and audio interaction in an attempt to extend the capabilities of modern medicine to rural settings [5]. Evidence has shown that the utilization of this real-time telemedicine can positively affect health outcomes involving myocardial ischemia and strokes [6]. Telemedicine has also been used during disaster medicine in response to various natural disasters, including earthquakes and hurricanes [7].

The US military developed several modalities of telemedicine for use in austere environments, ranging from video conferencing and store-and-forward technology to the application of virtual reality technologies to educational and clinical scenarios [8-9]. An important modality of this type is a version of the special medical augmentation response team (SMART).

This particular SMART team was a US army unit designed for short duration deployments and included the equipment necessary for audio, video, and document transmission in austere locations. This team set up low-bandwidth satellite equipment to help mobile army surgical hospital teams in austere environments utilize email and X-ray telemedicine services. While extremely helpful, this required a significant amount of equipment, specialized training, and staff. The drawbacks to this team were not insignificant; it required a technology specialist on site, a portable power generator, a color scanner printer, and specialized training for the deployment of the specific team [10]. Another telemedicine project, called “Operation Primetime 1,” utilized a similar set of equipment and connected two units in Macedonia with consultations to the Landstuhl Regional Medical Center in Germany, with significant success [11].

Positive impacts during various deployments to date, as well as evidence from myocardial ischemia and stroke data, show that telemedicine, if utilized correctly, can increase access to specialty care, improve health outcomes, and can avoid unnecessary evacuations [3-4,10-12]. However, current telemedicine systems have significant drawbacks, which include extensive training requirements, additional deployed support staff, and delayed access to expert medical advice and large, poorly mobile equipment.

This pilot feasibility study differs in that the video technology involved the use of a single helmet camera (GoPro) with real-time video, a phone and tablet with data/voice capability, a two-way radio, and no technology support specialists on site. This study sought to test a “plug-and-play system” that would fit in a small bag, where the deployed medical provider received audio feedback from the teleconsultant, and the consultant could visualize the condition of the patient in real time via a helmet camera. To date, there are no such systems available or studied. There is also scarce literature as to the use of real-time helmet cameras in the medical field.

The goal of the study was to determine the overall feasibility of a light-weight, portable, real-time video teleconsultation system. The secondary outcomes of this study were to determine the impact of a real-time, video feed teleconsultation on the completion of critical actions while caring for a simulated trauma patient, impacts on patient evacuations, and provider confidence at the point of care.

## Materials and methods

This was a double-blinded, within-subject crossover trial. The study took place in a level 1 trauma center with real patient rooms converted for simulation purposes. Subjects participating in the simulation comprised medics, medical students, and junior resident EM physicians acting as hands-on operators along with senior EM residents in their last year of training or staff EM physicians acting as teleconsultants. Informed consent was obtained for all 25 participating subjects.

**Figure 1:**
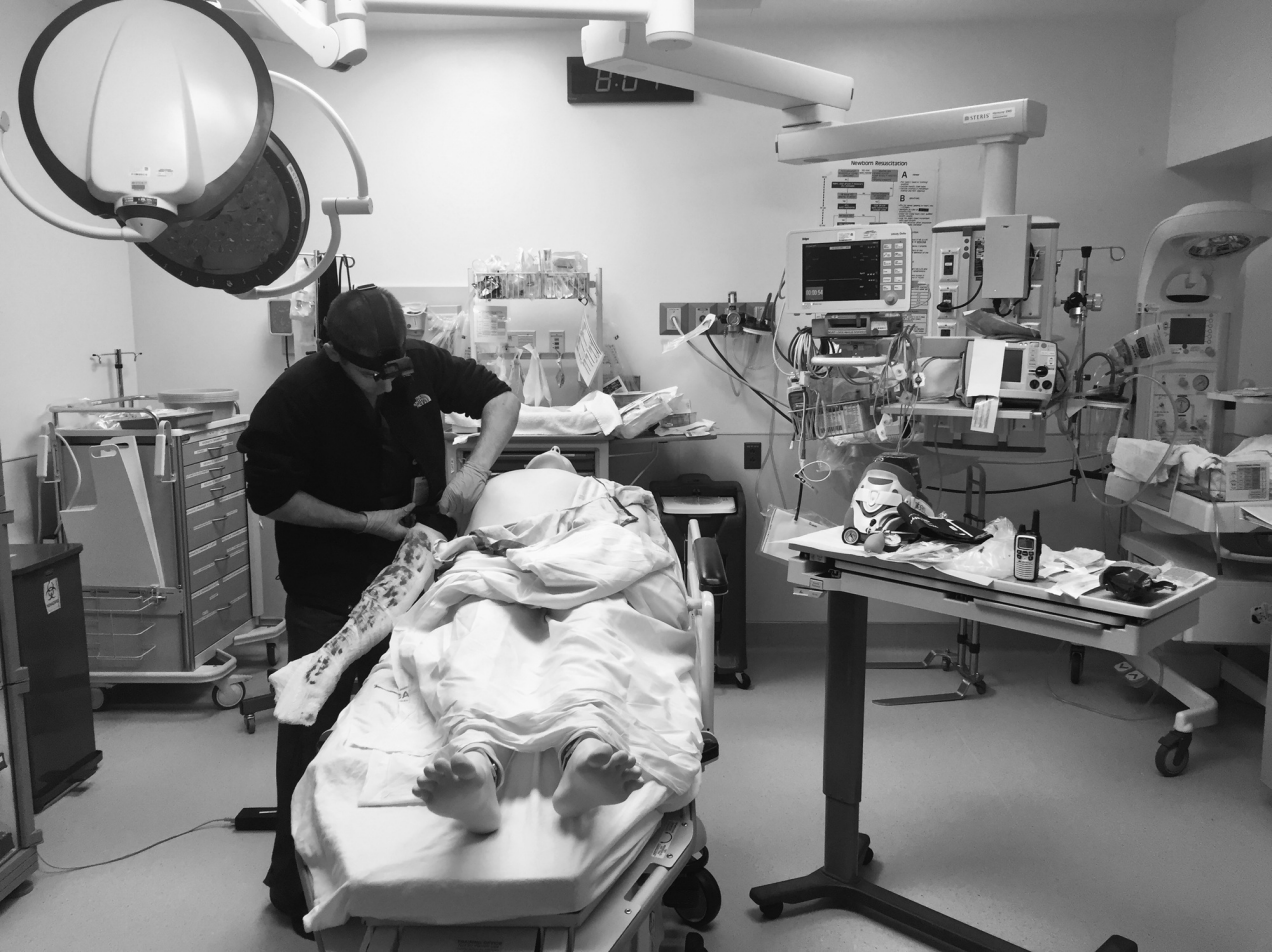
Operator in the simulated environment

Two similar simulation cases were created and involved commonly seen traumatic injuries, including an extremity injury with active hemorrhage and a thoracic injury causing a pneumothorax (Figure 1).

The operators completed the two simulation cases with a proctor in the room. All participants were allotted 10 minutes per case, at which point the simulation was ended. They had access to similar basic medical equipment, fluids, antibiotics, blood products, and ultrasound.

**Figure 2:**
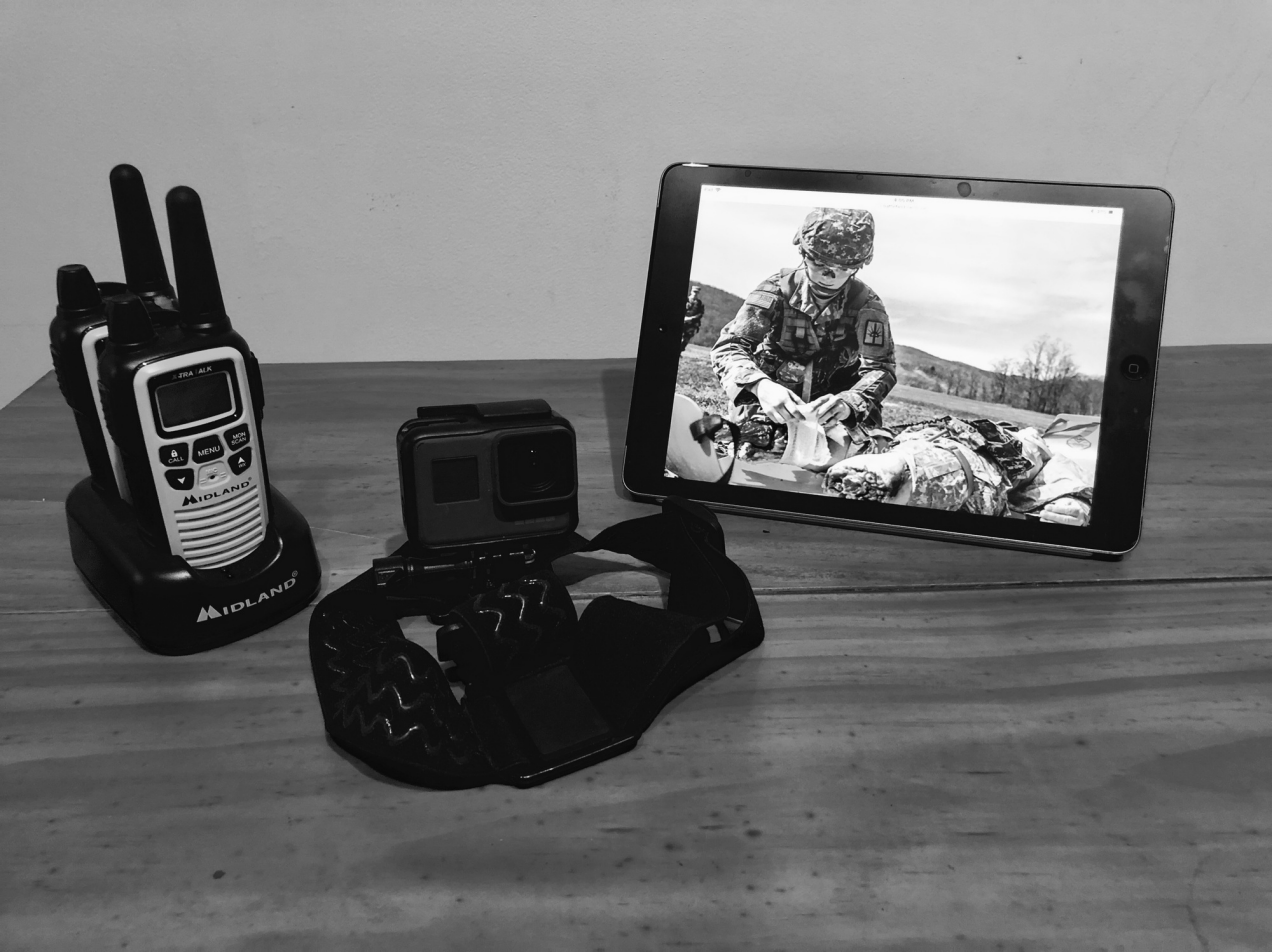
A two-way radio, camera, and monitor comprised the entire set-up

The two simulation rooms were not visible to the teleconsultant other than by the operator helmet video. The camera was set up at a wide point of view at 170 degrees with 4K resolution at 60 frames per second and utilized Bluetooth, along with either wireless or cellular signals for connectivity. The teleconsultant was given a portable tablet with a high definition screen. Image delay was less than one second and only limited by the hospital network or cellular data service. Both the camera and the tablet were dust-proof, shock-proof, waterproof, and had battery lives of four to eight hours depending on usage. The cost of the telemedicine equipment was under 1000 dollars (Figure 2).

The operators always had access to the teleconsultant by two-way radio. The intervention arm had the helmet-mounted camera transmitting real-time video to the teleconsultant. The operators always had the helmet camera attached and turned on but were not told if live images were being sent to a teleconsultant. The teleconsultant was advised that they could not tell the operator if they had a video feed during the case or ask them what they were seeing.

During the simulated cases, each operator was evaluated on various patient outcomes to include: time to identification of life threats, time to critical interventions, and time to evacuation decision. Participants were surveyed in various aspects of the intervention, including video quality, confidence in the technology, and confidence in decision-making. Data were analyzed by a statistician, blinded to the use of video, using the Cox-proportional hazards model.

## Results

**Figure 3:**
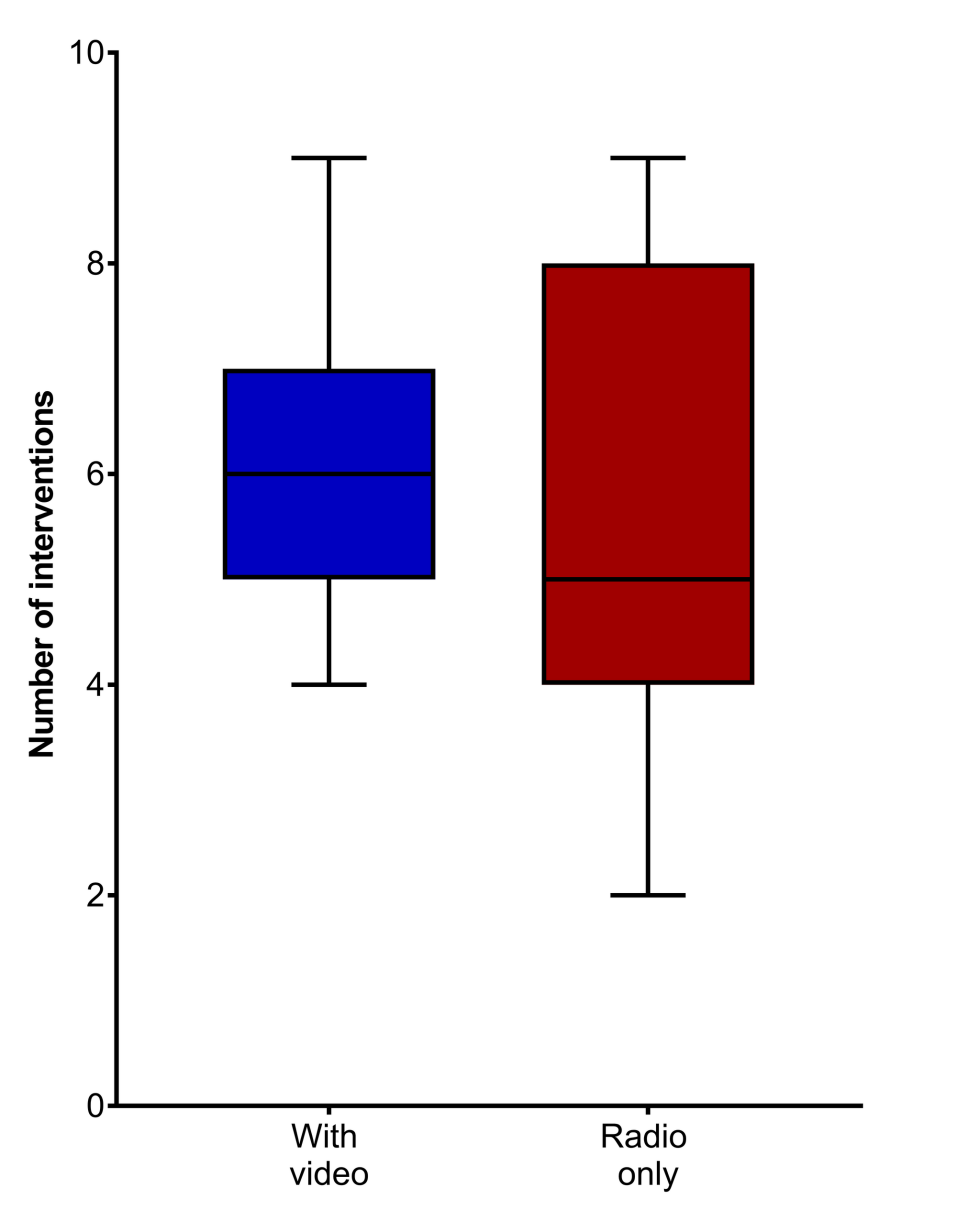
Number of interventions

**Figure 4:**
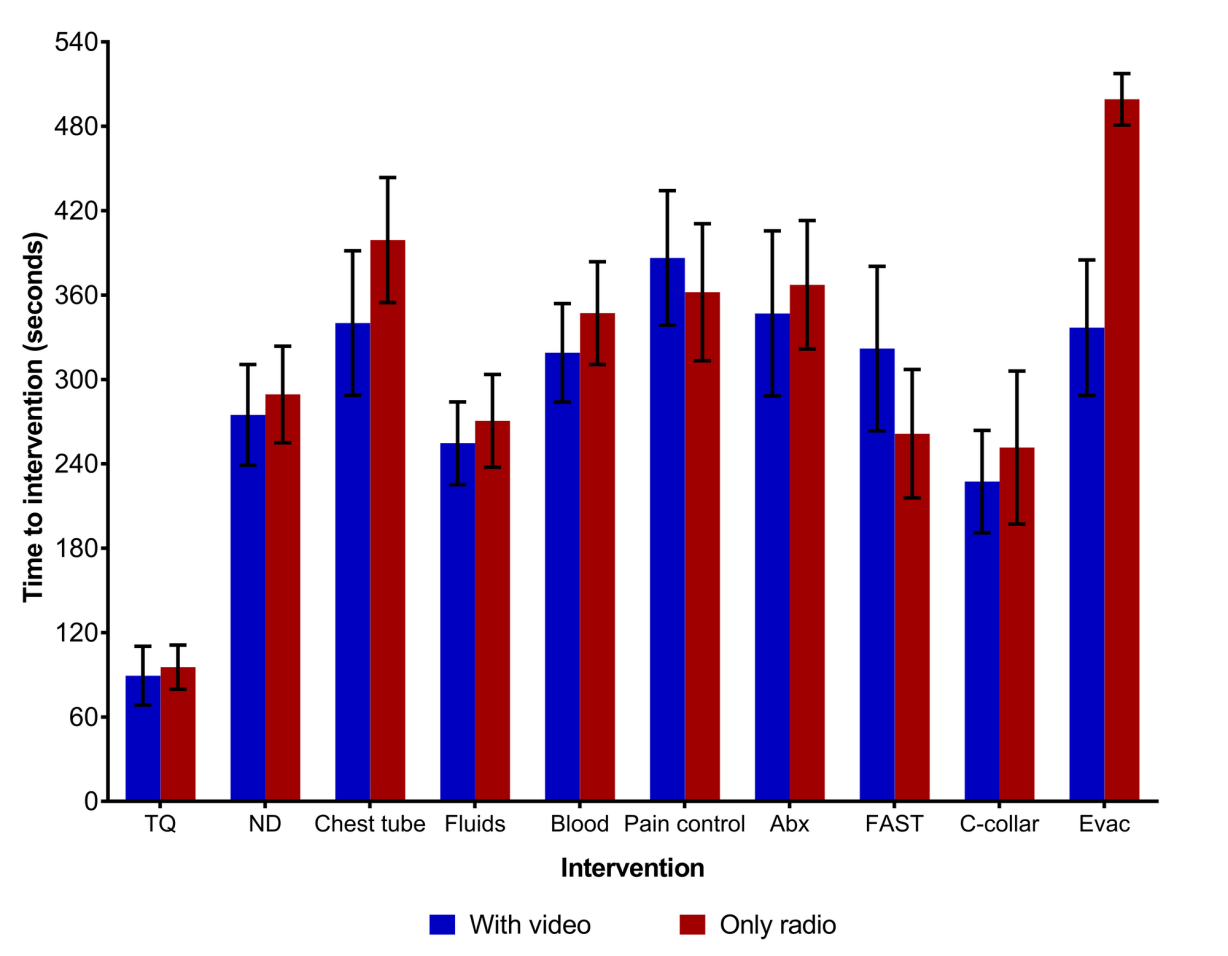
Time to intervention comparison TQ=Tourniquet, ND=Needle Decompression, ABX=Antibiotics, FAST=Focused Assessment with Sonography for Trauma, C Collar=Cervical Collar, Evac=Evacuation

There were no technical difficulties encountered during the simulated trauma cases. This included when the hospital wireless signal was not functional and the equipment ran solely on cellular data for video transmission. No significant difference was found with the addition of video as opposed to solely two-way radio communication in terms of the number of interventions or time to interventions (Figures 3-4).

There was a trend in most cases, favoring those with video to perform procedures faster and to evacuate more quickly. In cases without video, 14 patients were evacuated in the maximum allotted time (10 minutes), as opposed to 18 cases with video. This would imply a delay in evacuation when the teleconsultant had no video feed. The subgroup analysis based on operator level (medics vs. junior physicians) revealed no significant difference. There was no significant difference in operator or teleconsultant confidence in terms of patient care or procedures performed (p= 0.48 and p=0.37, respectively).

## Discussion

Telemedicine studies have demonstrated improved patient care, health outcomes, and confidence level of providers [3-4,10-12]. Although this study did not find any significant differences in critical actions with the addition of real-time video, it did demonstrate the overall feasibility and ease of use of lightweight, portable, real-time video technology for teleconsultation. Additionally, it could be operated with minimal specialized technological skills, training, or support personnel.

Of note, there were no technological difficulties during the study, and video clarity was not compromised even with a wireless network disruption. Portability was tested by utilizing both the hospital wireless network and cellular data for connectivity. It is envisioned that a system like this could be utilized now, with EMS services in areas that have 4G cellular connectivity. This could give the hospital-based medical team time to prepare for a sick patients' arrival or give additional medical guidance to EMS units. As global satellite technology continues to advance in speed and size of data transmission, it is easy to anticipate a similar system being utilized in austere settings, even with limited or poor cellular access. Further feasibility studies are required for this to become a reality.

There are multiple reasons that may have led to this studies lack of significant differences in patient outcomes. This was designed as a feasibility study and was limited in terms of the number of participants and not powered to look for significant differences in outcomes. The simulated injuries were very apparent and straightforward, which may not have challenged the operators enough, leading to less reliance on the teleconsultant. In addition, all of these operators had undergone tactical combat casualty care training within the past two years. This is a military course designed to prepare personnel for the initial phase of resuscitation [13]. As such, the subjects may not have required significant teleconsultation in our scenarios.

It was unclear from this study why the teleconsultants did not feel more confident with live video images of the simulation. Prior studies on telemedicine have shown that healthcare providers using telemedicine tend to believe it improves health outcomes and the care provided [14-16]. This may also have been because of the straightforward nature of the simulated injuries causing less interventional requirements from the telemedicine consultants.

Further study on this topic should include a larger subject group as well as more complex simulated injuries and illness. It may also be targeted towards prolonged field care, for example, intubated patients with multiple injuries and critical care needs in simulated, austere environments or with prolonged transport times. Additional study is also needed to examine the feasibility of this equipment in real-world settings, such as the real-time video feeds of traumatically injured patients prior to their arrival at trauma centers.

Limitations

As with many pilot studies, this study utilized a small group of subjects in a short time frame, limiting its power and external validity. Also, today’s live-feed video and wireless technology are changing rapidly such that in the time frame it took to complete the study, the camera used had already undergone two iteration changes by the manufacturer, with live-feed upgrades. The utilization of both wireless and cellular data to stream real-time video demonstrates this system’s remarkable adaptability but also limits its widespread use to areas with this type of data connectivity.

## Conclusions

Telemedicine will continue to be integrated into the healthcare system and help meet challenging medical needs. To date, most telemedicine systems require bulky, expensive, and stationary equipment. This study demonstrated the overall feasibility of using portable, lightweight, real-time video technology in teleconsultation. Further research on the portable, real-time video teleconsultation of acutely ill patients is needed to better assess how this resource could best be utilized in austere settings.
